# New Appliances and Things Medical

**Published:** 1904-06-11

**Authors:** 


### NEW APPLIANCES AND THINGS MEDICAL.

### ALBUMENISED HUMANISED MILK.

(Prepared by the Aerated Cream and Dairy Company, Limited, 21 Wellington Road, Battersea, London, S.W.)

This milk is supplied in small bottles containing about half a pint, and is intended for artificial infant feeding, being of such a nature that it may at once be gently warmed and added to the feeding bottle. Until opened the milk keeps perfectly, and after opening keeps as ordinary milk. The sample at present under observation was put up on March 25th, and is perfectly sweet and good. It contains no preservative. The milk is made up according to two formulæ, the object being to imitate as closely as possible the chemical composition of human milk. Subsequent to preparation it is treated by the patent process of the Aerated Milk Company. The maker of the milk has given us the fullest information with regard to it, and from the analysis to which it has been subjected it is evident that chemically the milk corresponds closely with average human milk. We regret that we cannot accept without further evidence the assumption that this chemical similarity or even identity is synonymous with physiological identity. No milk, prepared or otherwise, so far as we know, corresponds in digestibility with human milk, although much has been done and probably can still be done chemically to minimise the physiological difference between human and other milks. The preparation gives upon artificial digestion a full curd and may be regarded as a satisfactory substitute for human milk from the chemical side and should certainly be tried by the profession in those difficulties so often presented in infant nutrition. No trouble has been spared by the maker of this milk, and we hope further trial will accord to it the success which it unquestionably deserves.

